# Effects of FES‐Rowing Exercise on the Time‐Dependent Changes in Bone Microarchitecture After Spinal Cord Injury: A Cross‐Sectional Investigation

**DOI:** 10.1002/jbm4.10200

**Published:** 2019-09-03

**Authors:** Adina E Draghici, J Andrew Taylor, Mary L Bouxsein, Sandra J Shefelbine

**Affiliations:** ^1^ Department of Bioengineering Northeastern University Boston MA USA; ^2^ Cardiovascular Research Laboratory Spaulding Rehabilitation Hospital Boston MA USA; ^3^ Department of Physical Medicine and Rehabilitation Harvard Medical School Boston MA USA; ^4^ Endocrine Unit Massachusetts General Hospital and Harvard Medical School Boston MA USA; ^5^ Center for Advanced Orthopaedic Studies Beth Israel Deaconess Medical Center Boston MA USA; ^6^ Department of Mechanical and Industrial Engineering Northeastern University Boston MA USA

**Keywords:** BONE QCT/MICROCT, ANALYSIS/QUANTITATION OF BONE, OSTEOPOROSIS, DISEASES AND DISORDERS OF/RELATED TO BONE, EXERCISE, BIOMECHANICS, ORTHOPEDICS

## Abstract

Disuse osteoporosis is a serious, secondary consequence of spinal cord injury (SCI). Numerous pharmacological and exercise therapies have been implemented to mitigate bone loss after SCI. However, these therapies have not been shown to improve bone density, potentially because of insufficient duration and magnitude of loading and/or inability of imaging modalities to capture changes in bone microarchitecture. In this cross‐sectional study, we evaluated bone microstructure of the distal tibia and radius using HR‐pQCT in men with SCI (*N* = 13) who regularly trained with functional electrical stimulation‐ (FES‐) rowing. We aimed to determine whether the amount of FES‐rowing (total distance rowed and peak foot force) and/or time since injury (TSI) predict bone loss after SCI. We assessed volumetric density of the total, cortical, and trabecular compartments, cortical thickness, and trabecular thickness. Using linear regression analysis, we found that TSI was not associated with any of the tibial bone metrics. In fact, none of the variables (TSI, total distance rowed, and peak foot force) independently predicted bone loss. Using stepwise regression, when all three variables were considered together, we found a strong prediction for trabecular microstructure (trabecular vBMD: *R* ^2^ = 0.53; *p* = 0.06; trabecular thickness: *R* ^2^ = 0.72; *p* < 0.01), but not cortical bone metrics. In particular, trabecular vBMD and thickness were negatively associated with TSI and positively associated with distance rowed. Foot force contributed markedly less to trabecular bone than distance rowed or TSI. Our results suggest that regular FES‐rowing may have the capacity to alter the time‐dependent bone negative effects of SCI on trabecular bone density and microstructure. © 2019 The Authors. JBMR Plus published by Wiley Periodicals, Inc. on behalf of the American Society for Bone and Mineral Research.

## Introduction

Disuse osteoporosis is a serious secondary consequence of spinal cord injury (SCI).^1^, ^2^ Bone loss is directly related to time since injury (TSI)^3^; BMD decreases rapidly by about 40% in the first 3 years^3^, ^4^ and reaches a plateau by 7 years post injury.^5^, ^6^ The sublesional bone loss occurs primarily in the trabecular‐rich skeletal sites of the proximal tibia and distal femur,^1^, ^7^ greatly increasing risk of fractures.^8^ As a result, numerous pharmacological and mechanical therapies have been employed to prevent bone loss after SCI.

The pathogenesis of osteoporosis after SCI is multifactorial resulting from hormonal changes, neuronal mechanisms,^9^ autonomic denervation^10^ below injury level, and to a great extent from disuse.^11^ The loss of muscle contractions and weight‐bearing results in a lack of mechanical loading and subsequent bone loss. Bone remodels continuously in response to applied stresses and strains, and these adaptations are central to maintain bone mass and ensure sufficient bone strength. However, interventions that load sublesional bone in SCI (eg, standing frame, body‐weight‐supported treadmill walking, functional electrical stimulation‐ [FES‐] cycling) have rarely been shown to have even a modest effect,^12^, ^13^ if any effect at all.^13^, ^14^, ^15^, ^16^, ^17^, ^18^, ^19^, ^20^, ^21^, ^22^, ^23^, ^24^, ^25^ This could be because of insufficient loading intensity (magnitude, number of cycles, and duration) to promote changes in bone density. In fact, very few studies quantify the intensity parameters used in their interventions. One of the few studies that did quantify load found that plantar flexor stimulation at loads of 150% body weight (BW) increased tibial BMD, whereas loads of 40% BW were ineffective.^26^, ^27^ Another factor that could account for the apparent ineffectiveness of loading interventions is insufficient imaging resolution to detect changes in bone microarchitecture. Dual‐energy X‐ray absorptiometry (DXA) has insufficient resolution to offer information regarding volumetric changes in bone mineral content, or to assess changes in trabecular and cortical microarchitecture at the proximal tibia and distal femur, the sites of highest risk fracture in those with SCI. On the other hand, high‐resolution peripheral quantitative computed tomography (HR‐pQCT) has unique capabilities to quantitatively assess volumetric bone mineral density (vBMD), as well as geometrical and microstructural cortical and trabecular features.^28^, ^29^ Both cortical and trabecular structures contribute to wholebone strength, but respond differently to loading. Thus, HR‐pQCT may reveal previously undetected adaptations and differences in trabecular and cortical microstructure resulting from mechanical loading interventions.

FES‐rowing actively engages the leg muscles and dynamically loads the bones^30^, ^31^, ^32^, ^33^; hence, FES‐rowing may alter the time‐dependent tibial bone loss seen after injury. We used HR‐pQCT to assess bone microarchitecture cross‐sectionally in men with SCI who had regularly trained with FES‐rowing. Because bone is mechanoadaptive, we aimed to determine whether the amount of FES‐rowing exercise and/or TSI were predictive of bone density. We hypothesized that across these individuals, both amount of exercise (distance and magnitude) and TSI would predict bone density and microarchitecture.

## Subjects and Methods

### Patient recruitment

We recruited 13 men (mean age: 31.9 ± 9.96 years; range 20 to 54 years) from current participants in the Spaulding Rehabilitation Hospital SCI Exercise program who had regularly trained with FES‐rowing. These individuals represented a cross‐sectional cohort across a range of TSI (9 to 101 months), providing data to assess the effect of FES‐rowing exercise on microarchitecture degradation across the time range with greatest bone loss. Volunteers were wheelchair‐dependent and had a SCI of American Spinal Injury Association (AIS)^34^ A to C at the neurological level C4 to T8. All participants were medically stable and able to tolerate electrical stimulation without dysreflexia. No participant had suffered lower limb fractures and no participant was treated for osteoporosis at any point in time prior to this study. All procedures were approved by the Institutional Review Board at Spaulding Rehabilitation Hospital and written informed consent was obtained from all participants.

### Protocol

#### Rowing history

For all participants, we obtained their FES‐rowing exercise history, which included time of initiation of FES‐rowing relative to injury and duration of FES‐rowing (Table 1). Intensity of FES‐rowing was characterized by magnitude (ie, average peak foot force) and distance (ie, total distance rowed; Table 1). Peak foot force for each volunteer was measured at the beginning of a typical FES‐row training session on an adapted instrumented Concept2 ergometer (Concept2, Morrisville, VT, USA). The instrumentation and measurements obtained have been described in detail in our previous work.^35^ Briefly, forces at the feet were measured using button‐style compression load cells (Strain Measurement Devices Inc., Wallingford, CT, USA, SMD4856, range 0 to 980 N; accuracy 0.05%) mounted under the toe and heel of each foot. Forces were recorded at 100 Hz. The peak foot force of the leg that underwent imaging was determined as the average peak foot force during approximately 5 min of FES‐rowing (MATLAB R2014b; The MathWorks, Natick, MA, USA). Our group has previously shown that peak foot force in those with SCI remains constant even with increased intensity of FES‐rowing.^35^ Thus, the average peak foot force over 5 min of a regular FES‐rowing session was considered characteristic of the magnitude of loading experienced by each subject throughout FES‐rowing training. Total distance rowed represented the FES‐rowing distance in meters over the duration of training.

**Table 1:** Demographics, Injury Information, and FES‐Rowing History for Study Participants

Demographics (*n* =13)	Mean ± SD
Age (years)	32 ± 10
Height (m)	1.8 ± 0.7
Weight (kg)	80 ± 16
Race/ethnic origin	69% White; 15% Black; 8% Asian; 8% Hispanic
Injury health history	
Injury level	60% C4 to C8; 40% T1 to T8
ASIA	46% A; 31% B; 23% C
TSI (years)	3.3 ± 2.2 (range 0.8 to 8.4)
TSI when started FES‐rowing (years)	1.6 ± 1.6 (range 0.3 to 6.1)
Duration of FES‐rowing (years)	1.7 ± 1.5 (range 0.15 to 4.6)
FES‐rowing history	
Total distance rowed (km)	462 ± 478 (range 8 to 1790)
Peak foot force (N)	190 ± 93 (range 90 to 440)

All values are expressed as mean ± SD.

FES = functional electrical stimulation; ASIA = American Spinal Cord Injury Association Impairment Scale; TSI = time since injury.

#### High‐resolution peripheral quantitative‐CT

Scans were performed at the Bone Density Center at Massachusetts General Hospital using a HR‐pQCT scanner (XtremeCT, Scanco Medical, Brüttisellen, Switzerland). Scans of the nondominant side were obtained at the distal radius and distal tibia (60 kVp, 95 mAs and 126 mm field of view), as previously described.^28^, ^36^, ^37^, ^38^, ^39^, ^40^ Scans were acquired beginning at 9.5 mm and at 22.5 mm proximal to the endplate of the distal radius and distal tibia, respectively. The total volume of the scanned region included 110 slices over a 9 mm region, with an isotropic voxel size of 82 µm. Each scan duration was 2.8 min with an effective radiation dose of 3 µSv. Volumetric density and trabecular bone microarchitecture were obtained using the standard evaluation protocol, whereas cortical bone microarchitecture was derived from an extended cortical analysis.^41^, ^42^ We used the following parameters to characterize trabecular and cortical bone microarchitecture: volumetric density of the total, cortical, and trabecular compartments; cortical thickness; and trabecular thickness. Ideally, we would have obtained bone microarchitecture at the distal femur and proximal tibia, the sites with the highest rate of bone loss and fracture risk in the SCI population. However, the HR‐pQCT scanner is restricted to the ultradistal sites of the appendicular skeleton, such as the distal tibia and distal radius. Nonetheless, several studies have shown that bone density and microstructural parameters measured by HR‐pQCT of the distal radius and tibia reflect overall strength of the central skeleton,^43^, ^44^ and in fact, distal tibia vBMD assessed by HR‐pQCT is comparable to proximal femur vBMD assessed by cQCT.^43^ Thus, microarchitecture of the distal tibia is likely comparable to that of the proximal tibia and distal femur.

### Statistical analysis

We used linear regression analysis to assess the relationship between bone metrics (at the distal radius and distal tibia) and the following independent variables: TSI, total distance rowed, and peak foot force. The radius was measured to examine bone density at a site less affected by SCI and it was used to confirm that only sublesional bone demonstrated loss related to TSI. To determine if there were combined effects or significant interactions, we also performed a stepwise linear regression analysis with the above variables. The stepwise regression is a forward/backward model selection approach in which all independent variables (ie, TSI, total distance rowed, peak foot force, and interactions) are considered initial predictors of bone metrics. This provides a systematic search of models, where each new model is obtained by iteratively adding or subtracting variables based on their predictive power. It should be noted that with *n* = 13 and three predictors, the statistical power would be sufficient (>80%) at a *p*‐value of 0.05 when the regression has *R* ^2^ above 0.56. The contribution of each variable independent of absolute value and/or unit was assessed using *Z*‐score coefficients. Statistical significance was set at *p* ≤ 0.05. Statistical analyses were performed using RStudio for Linux (RStudio Team 2016, Integrated Development for R; RStudio Inc., Boston, MA, USA).

## Results

The majority of volunteers (11 out of 13) had started FES‐rowing within 2 years of injury. At the time of study, all participants had been FES‐rowing for at least 2 months and up to 5 years and training regularly between 1 to 3×/week. Across the 13 participants, the total FES‐rowing distance performed ranged widely, from 8000 meters (approximately 2 months of training) to 1800 km (approximately 5 years of training). Because leg loading is influenced by the extent of muscle atrophy and muscle extensor tone, peak foot force also ranged widely from approximately 90 N to approximately 450 N, corresponding to a range of 15% BW to approximately 50% BW. Though most studies of joint loading in gait report load as % BW, in those with SCI the peak foot force during FES‐rowing is not related to BW; thus, we report force in Newtons. A summary of the participants’ rowing characteristics is provided in Table 1.

### Association between bone microarchitecture and exercise intensity

**Figure 1:**
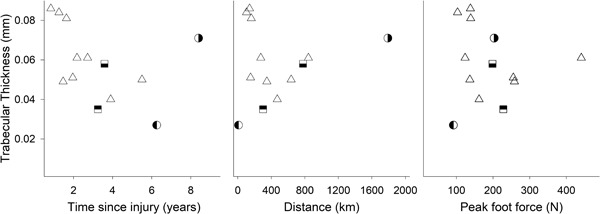
Tibial trabecular thickness as a function of time since injury (TSI), distance, and peak foot force. The half‐filled symbols represent different individuals who exemplify the interacting effects of TSI, distance, and peak foot force. Individuals with similar TSI but greater distance rowed have greater trabecular thickness.

**Figure 2:**
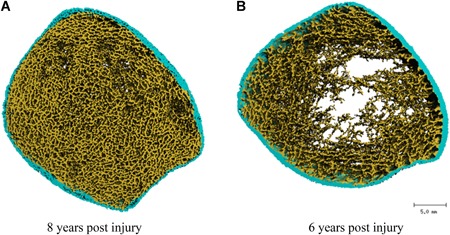
3D reconstruction of HR‐pQCT scans of the distal tibia in two representative spinal cord injury individuals: (*A*) 8 years postinjury and (*B*) 6 years postinjury. Note that these two subjects are represented by half‐filled circles in Fig. 1.

For the radius, neither TSI nor any exercise variable predicted any bone metric (*R* ^2^ < 0.15; *p* > 0.05). In addition, stepwise regression indicated that TSI and exercise variables had no combined effects or significant interactions (*R* ^2^ < 0.15; *p* > 0.05). Those with high‐level injuries had lower radius bone density compared with those with low level injuries. For the tibia, in contrast to the well‐established time‐dependent bone loss, TSI did not predict any bone metric (*R* ^2^ < 0.15; *p* > 0.05; Fig. 1). In fact, none of the variables (TSI, total distance rowed, and peak foot force) considered independently predicted bone loss post injury (*R* ^2^ < 0.18; *p* > 0.05; Fig. 1). The stepwise regression indicated that TSI and exercise variables had no combined effects or significant interactions for cortical microstructure (cortical vBMD and cortical thickness) or total vBMD (all *R* ^2^ < 0.35; all *p* > 0.05). However, for trabecular bone, when the three variables were considered together, there were combined effects, but no significant interactions. For trabecular vBMD, the prediction approached significance at *R* ^2^ = 0.53 (*p* = 0.06, power of 77%), and for trabecular thickness, the combination of all three variables was strongly predictive at *R* ^2^ = 0.72 (*p* < 0.01). *Z*‐scores for each independent variable showed that TSI and total distance rowed had similar but opposite effect on trabecular vBMD (TSI: –1.1, total distance rowed: 1.1), whereas force contributed less (–0.5). This was also the case for trabecular thickness; TSI and total distance rowed had similar magnitude, but opposite contributions (TSI: –1.3, total distance rowed: 1.2), with peak foot force contributing less (–0.7). These results are exemplified in Fig. 1, showing trabecular thickness as a function of TSI, total distance rowed, and peak foot force. The participant with the greatest TSI (approximately 8 years; right half‐filled circle; Fig. 2a) had one of the highest values for trabecular thickness. However, this individual had the greatest volume of FES‐rowing (distance), with a relatively average peak foot force. In contrast, another participant with similar TSI (approximately 6 years; left half‐filled circle; Fig. 2b) had the lowest trabecular thickness, which was associated with the smallest distance rowed and the least peak foot force. Likewise, two participants with similar TSI (approximately 3 years; half‐filled squares) and peak foot force (approximately 200 N) demonstrated different values for trabecular thickness because of the difference in rowing distance.

## Discussion

Our results suggest that FES‐rowing exercise may alter the time‐dependent bone loss seen after SCI. Surprisingly, the well‐established time‐dependent bone loss in the SCI population was not seen in our cohort that regularly trained with FES‐rowing. In this population, we found that bone microarchitecture degradation could be predicted only if we considered both TSI and amount of exercise performed. Even though bone declines rapidly post injury, our results show that the trabecular microstructure deterioration may in fact be altered with greater intensity of FES‐rowing. Importantly, the potential effect of FES‐rowing must be considered in the context of the “intervention”; these volunteers were not part of an exercise training study or a clinical trial, but had simply enrolled in an exercise program that offers this form of exercise. Moreover, the volunteers were not instructed on a specific training regime or duration, the amount of exercise being individually chosen. This was to our advantage as we had a broad range of distance rowed and loading force, which enabled correlations to be made. It should also be considered that most of our participants (11 out of 13) started FES‐rowing in the first 2 years post SCI, when bone density declines most rapidly, but still possesses the ability to adapt to mechanical loading. Future studies are required to determine if FES‐rowing, and exercise therapy in general, is most effective in maintaining rather than increasing bone mass.

Most studies have failed to find an effect of various exercise loading protocols on leg bone in SCI.^13^, ^14^, ^15^, ^16^, ^17^, ^18^, ^19^, ^20^, ^21^, ^22^, ^23^, ^24^, ^25^ However, our results support the few previous studies that found high exercise volume (magnitude × frequency) effective in improving bone health. FES‐cycling resulted in an increase in trabecular vBMD in the distal femur, but no effect in the cortical bone or tibia.^12^ Cyclic FES stimulation of the quadriceps up to 1.5 BW resulted in an increase in distal femur BMD, whereas a 0.4 BW static load was insufficient to alter bone density.^27^ Moreover, FES‐rowing in a single individual with chronic SCI who had regularly trained for more than 12 years apparently provided sufficient loading to result in greater tibial bone metrics compared with FES untrained chronic SCI.^31^ Similar to our results, these previous studies found a positive effect of cyclic loading exercises on trabecular microstructure, with no effect on the cortical compartment density.^27^, ^31^ This differentiated response to loading might be attributed to trabecular bone being more metabolically active based on a high surface‐to‐bone ratio with prime access to bone progenitors in the marrow.

The lack of an effect found by previous exercise studies might be because of the imaging modalities used to assess bone density. One of the few studies that investigated the effect of cyclic FES stimulation on bone density only found an effect in the femur and not the tibia across the subject population when assessed by pQCT, but found an effect on tibial trabecular microstructure when assessed by high‐resolution CT imaging in a single subject.^27^ This underscores the importance of high‐resolution imaging modalities that can evaluate bone microstructure. In particular, DXA‐BMD measurements are unable to discern trabecular and cortical bone and would have been likely insensitive to the compartment‐specific effects seen in the current study.

We investigated the distal tibia based on the restrictions of the HR‐pQCT scanner to ultradistal sites of the appendicular skeleton. Although the primary sites of bone loss and fracture risk in those with SCI are the distal femur and proximal tibia, the distal tibia is the region closest to the external loading resulting in FES‐rowing. However, the distal femur and proximal tibia may be exposed to even greater loads than the distal tibia because of the internal muscle loads of the stimulated muscles crossing the knee joint. Future work will examine the primary sites of fracture around the knee joint using flat panel volume CT.

Surprisingly, although peak foot force provided significant predictive power for trabecular microstructure, the coefficient was negative, suggesting that greater force relates to lesser trabecular bone. This is counterintuitive and in contrast to extensive evidence in both animals and humans.^12^, ^27^, ^45^, ^46^ However, the stepwise linear regression found that peak foot force had the least predictive power and only contributed significantly when considered together with TSI and distance rowed. The apparent “negative” effect of foot force on trabecular microstructure might be understood in the context of the individuals who were within 2 years of injury and had similar trabecular bone metrics, yet a wide range in peak foot force (see Fig. 1). The stepwise regression found the best model for trabecular bone metrics “normalized” foot force via a negative coefficient. Hence, it is unlikely that foot force itself had a negative effect on trabecular microarchitecture; the best explanation is that it simply provided further statistical predictive power.

The lack of a control group of individuals with SCI that are not FES‐rowing is a limitation of this study. The inclusion of nontrained SCI individuals would have allowed a more direct characterization of the effect of FES‐rowing on bone microarchitecture. However, the well‐established time‐dependent bone degradation^6^ is not present in our SCI cohort, indicating the potential protective effect of FES‐rowing on bone loss. The heterogeneity of the study population could be considered a potential limitation of this study. Our cohort included individuals with a wide age range (approximately 20 years to approximately 50 years) and across levels of injury from C4 to T8. However, age was not a predictor for radial bone and those with high‐ and low‐level SCI did not differ in TSI, total distance rowed, and peak foot force. Thus, despite the heterogeneity of the population, our results indicate that FES‐rowing may have a protective effect for bone microstructure. It should be considered that most of our subjects (11 out of 13) had started FES‐rowing within 2 years of SCI when a plateau in bone loss has not yet been reached and bone can still adapt to mechanical loading. FES‐rowing may not have similar effects on bone in those with chronic SCI (>2 years) who have already lost significant bone mass. We used the heterogeneity of the subjects to our advantage by considering the contribution of influencing parameters (such as TSI, distance rowed, and peak foot force) to bone microarchitecture measures, which could not be done if the data were more homogenous. These results inform the design of future studies by indicating that FES‐rowing may be most effective when started soon after injury and when exposure is maximized, and that the magnitude of foot force may have only a modest role in adaptations.

Our results suggest that FES‐rowing exercise may have the capacity to alter the relationship between bone loss and TSI after SCI. FES‐rowing of sufficient exposure could be able to prevent the extensive deterioration of the trabecular microarchitecture that occurs postinjury. Indeed, this was observed outside a controlled therapeutic setting and in an individualized community exercise program. Taking advantage of the bone sensitivity to load in the acute stages post injury using FES‐rowing might open new avenues to prevent and treat disuse osteoporosis in those with SCI. Future studies should be focused on determining if FES‐rowing, and exercise therapy in general, is most effective in maintaining rather than increasing bone mass.

## Disclosures

Adina E Draghici, J Andrew Taylor, Mary L Bouxsein, and Sandra J Shefelbine declare they have no conflict of interest.
